# “A Man With a Loving Heart”: A Systematic Review of Male Involvement in Cervical Cancer Screening in Africa

**DOI:** 10.3389/ijph.2024.1607447

**Published:** 2024-10-10

**Authors:** Teluleko N. Maseko, Joyce M. Tsoka-Gwegweni, Xolisile Dlamini

**Affiliations:** ^1^ Eswatini Ministry of Health Cancer Unit, University of Free State Faculty of Health Sciences Bloemfontein, Bloemfontein, South Africa; ^2^ Office of the Dean, Faculty of Health Sciences, University of the Free State, Bloemfontein, South Africa; ^3^ Eswatini Ministry of Health Cancer Unit, Mbabane, South Africa

**Keywords:** male involvement, cervical cancer, women health, Africa, screening

## Abstract

**Objective:**

This review assessed men’s involvement and the predictors of their involvement in cervical cancer screening.

**Methods:**

We conducted a comprehensive search of the following electronic databases: PubMed, EMBASE, CINAHL, African, Web of Science, and Scopus. The search was limited to articles published between January 2010 and January 2023.

**Results:**

A total of 17 studies met the inclusion criteria and were included for this review. More than 50% of the studies emphasised the relevance of male involvement in cervical cancer prevention in Africa and demonstrated the critical importance of this as a strategy.

**Conclusion:**

The results revealed that a significant proportion of male participants had a restricted comprehension of cervical cancer. Nevertheless, they expressed their willingness to provide backing for cervical cancer screening contingent upon their informed consent.

## Introduction

Although cervical cancer is a preventable and treatable disease, it ranks as the fourth most prevalent form of cancer among women worldwide [1]. The prevalence of this burden is most pronounced in sub-Saharan African nations, where age-standardised incidence rates range from 75 per 100,000 women in countries with the highest risk to less than 10 per 100,000 women in countries with the lowest risk [1]. According to available data from 2018, the Sub-Saharan area accounted for nearly 90% of recorded fatalities [2]. Differences in the rates of cervical cancer and deaths in different parts of the world are due to differences in social and structural factors that are linked to the disease, as well as differences in the information available about it, how to avoid getting it, how to get screened, and how to obtain good cancer treatment facilities. These disparities highlight regions that require targeted interventions to address the pressing needs of this area [3]. The global strategy proposed by the World Health Organization (WHO) aims to expedite the eradication of cervical cancer. This strategy envisions a world where cervical cancer is no longer a public health concern. To achieve this, the strategy emphasises the importance of implementing measures that are responsive to the specific needs of women, taking into account their social circumstances, as well as the various personal, cultural, social, structural, and economic barriers that impede their access to healthcare services.

Cervical cancer screening and treatment in Africa face numerous challenges, including a lack of awareness among women about potential risks and the need for screening procedures, geographical distance between healthcare facilities and rural populations, financial constraints, and cultural stigma. The financial burden of screening and treatment, including Pap tests and treatment, was also significant. Substandard healthcare facilities in many regions also pose challenges as they lack the necessary equipment and personnel to provide optimal quality cervical cancer care [1].

Human papillomavirus (HPV) infections that last for a long time, with one or more high-risk oncogenic forms, are the main cause of cervical cancer and carcinoma. Human papillomavirus (HPV) changes the epithelial cells in the transformation zone of the cervix, which is noticeable because it interferes with normal bodily functions [4]. Cervical cancer is a unique type of cancer characterised by an extended precancerous phase that spans several years prior to the onset of invasive malignancy. This extended period offers a significant opportunity for the identification and implementation of effective diagnostic and treatment strategies [5]. Cervical cancer is a malignant condition that can be effectively screened for. The purpose of screening is to detect cellular alterations in the cervix that have the potential to develop into cervical cancer if left untreated [6].

The efficacy of cervical cancer screening in reducing the occurrence of cervical cancer is well established. However, it is important to acknowledge that several variables might affect the rate at which individuals choose to undergo screening [7]. According to the literature, factors such as an individual’s perspective, cultural background, and attitude of their partners all affect how frequently people participate in cervical cancer screening programmes [8]. Multiple studies have shown that a significant number of women, especially those who lack male participation and possess little awareness about cervical cancer and screening, may not fully comprehend the advantages of undergoing screening in comparison to the potential repercussions of abstaining from it [9].

Africa has several preventive, treatment, and rehabilitation options for cervical cancer, although there are some limitations. These strategies include risk assessments, screening procedures, and therapeutic treatments. However, the use of these services remains limited owing to cultural and behavioural obstacles. References [10, 11] are provided. To optimise the effectiveness of cervical cancer screening and treatment initiatives, it is essential to have a comprehensive understanding of the male-related variables that impact the use of screening services among eligible women as well as their prevalence throughout the population. Therefore, in this review, we aimed to i) provide substantiation for decision-making and policy interventions that might enhance programmes and initiatives targeted at eradicating cervical cancer in Africa. ii) examine African men’s knowledge toward supporting their female partners inf cervical cancer screening.

## Methods

### Literature Search

This systematic review followed the PRISMA review guidelines and included peer-reviewed research publications (as described by [12]) on the national CC screening initiatives of Eswatini that appeared in English between 2010 and 2023. To find relevant research, the following five databases were searched: Web of science, CINAHL, Scopus, PubMed and EMBASE.

### The Search Strategy: Search Words

1. (Cervical cancer [MeSH] OR Cervical Neoplasm* OR Cervix Neoplasm*OR Cervix Ca* OR Cervical Ca* OR “Screening uptake” OR “Screening utilization” OR “Male/Men involvement” OR “Male support”
2. (Screening [MeSH] OR Early Detection OR Cancer Screening OR Early Diagnosis OR Cervical Cancer knowledge OR Screenings OR HPV OR HPV vaccine
3. “Africa” OR Sub-Saharan Africa OR Uganda OR Angola OR Benin OR Botswana OR Burkina Faso OR Burundi OR Cameroon OR Central African Republic OR Congo OR Côte d'Ivoire OR Djibouti OR Equatorial Guinea OR Zimbabwe OR Eritrea OR Ethiopia OR Gabon OR Gambia OR Ghana OR Guinea OR Guinea-Bissau OR Kenya OR Lesotho OR Liberia OR Madagascar OR Malawi OR Mali OR Mauritania OR Mauritius OR Mozambique OR Namibia OR Niger OR Nigeria OR Réunion OR Rwanda OR Sao Tome OR Principe OR Senegal OR Seychelles OR Sierra Leone OR Somalia OR South Africa OR Sudan OR Swaziland OR Tanzania OR Togo OR Zambia
4. # 1 AND # 2 AND #3

### Inclusion and Exclusion Criteria

Study designs that satisfied the following requirements were accepted: (i) quasi-randomised controlled trial and observational studies (i.e., cross-sectional, case-control, cohort); (ii) research performed in Africa between January 2010 and December 2023; and (iii) English-language publications. Studies with significant missing data were removed, as were case reports, case series, expert comments, studies done out of Africa, publications in languages other than English, qualitative research, and duplicate publications.

### Study Selection and Data Extraction

The articles discovered were imported into Covidence [13]. Following the exclusion of duplicate studies, two researchers analysed the abstracts and titles of papers to determine their relevance to the chosen review subject. The reviewers’ unanimity led to the establishment of an agreement. Subsequently, a comprehensive evaluation was conducted on the entire texts of the remaining articles to determine their suitability for continued inclusion. Ultimately, the two reviewers successfully retrieved all the pertinent materials.

### Quality Assessment of Included Studies

Two reviewers used the Newcastle-Ottawa Scale (NOS) for cross-sectional research to assess the quality of the selected papers [14]. This instrument has three distinct areas: selection, comparability, and results. Each category was evaluated on a scale of five stars, indicating the highest level of achievement. In the context of this review, the categorization of the studies was based on their respective ratings. Studies were deemed “excellent” if they achieved a score of 5 stars or more, “very good” if they obtained 4 stars, “good” if they received 3 stars, and “unsatisfactory” if their rating fell between the range of 0 and 2 stars. Supplementary Table S2 provide information on the quality and country of the included studies.

## Results

### Characteristics of the Studies

**FIGURE 1:**
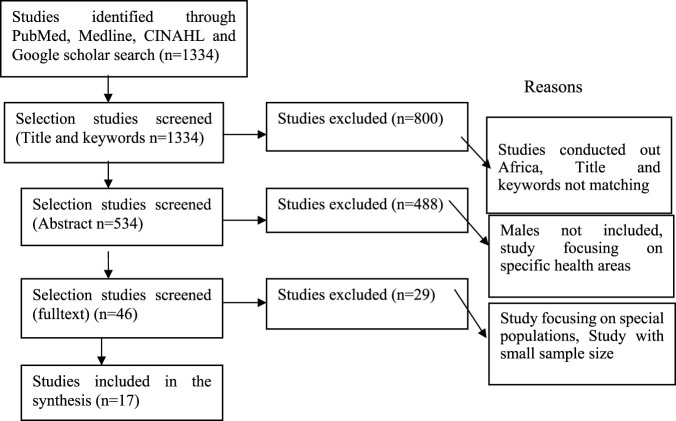
Prisma follow diagram for predictors of cervical cancer screening, January 2010 to December 2023, Africa.

A comprehensive search strategy yielded a total of 1334 studies that were obtained using both database and manual searching methods. After the removal of duplicate articles, 46 full-text articles were evaluated to determine their suitability for further analysis. In conclusion, 17 articles were included in this study see Figure 1. This review included a diverse sample size, including both male and female individuals. This review included research conducted in several sub-Saharan African nations: one study in Zambia, one study in Eswatini, one study in Malawi, three studies in Nigeria, three studies in South Africa, two studies in Kenya, three studies in Uganda, two studies in Ghana, and one study in Senegal. The bulk of the studies included in the analysis were conducted using a cross-sectional study design. A total of 28 studies were assessed to have a quality score of “very good” (5 stars), while one research study had a quality value of “good” (4 stars).

### Predictors of Cervical Cancer Screening

Research conducted in Uganda, South Africa, and Nigeria has revealed a notable correlation between insufficient information about HPV and cervical cancer screening and reduced usage of cervical cancer screening services [15–19]. In contrast, prior research by four studies [18, 20–22] showed that cultural values play a significant role in predicting the adoption of screening services. According to previous studies [23–26], the implementation of screening programmes in Kenya, Zambia, and Malawi has a positive impact on the uptake of screening services. Uganda saw a notable enhancement in the awareness of cervical cancer screening [19]. This improvement was observed after the implementation of educational workshops targeting males, aimed at equipping them with proper information pertaining to cervical cancer and the importance of screening. These workshops not only increased knowledge among males, but also encouraged them to support and encourage women in their lives to undergo screening. Consequently, more women in Uganda have been accessing and utilising cervical cancer screening services, leading to a higher overall adoption rate. Similarly, two studies (16, 27) documented a positive correlation between higher levels of education and increased usage of screening services. Several factors have been shown to decrease the chance of women undergoing cervical cancer screening, including decision-making, perceived vulnerability, reduced sexual activity within marriage, stigma, and the screening procedure [16, 20, 21, 23, 25, 26]. The association between cervical cancer screening uptake and factors such as HPV (28, 30), limited understanding of the gravity of cervical cancer (14, 17, 25), and support from partners (29) was statistically significant (Table 1).

**TABLE 1:** Characteristics of the included studies from January 2010 to December 2023, Africa.

Author	Publication year	Country	Research question/aim of the study	Sample size	Study design	Predictors	Quality score (stars)
Chigozie et al [27]	2021	Nigeria	The study looked into Nigerian men’s willingness to encourage and pay for family member to obtain HPV vaccine and cervical screening	352 men	Quantitative Cross-sectional	Perception of HPV risk Encourage and pay for service Education level Residency Income	8
Rwamugira et al [28]	2019	South Africa	The study aimed at empowering men to teach their female partners and family members about cervical cancer and motivate them to be screened	120 men	Interventional Cross-sectional	HPV infection Culture Education level Knowledge about cervical cancer	7
Maree et al [29]	2011	South Africa	The purpose of the study was to determine the level of knowledge regarding cervical cancer among Black men living in Ga-Rankuwa, South Africa	980 men	Quantitative Cross-sectional	Education level Partner support Ever screened Community beliefs	8
Rwamugira et al [30]	2019	South Africa	The purpose of the study was to describe the knowledge men, living in Muldersdrift, had about cervical cancer, cervical cancer screening and the cervical cancer screening programme and how they preferred to be taught about these health issues	120 men	Quantitative Intervention research	Outcomes of educational programme Men’s knowledge Culture Men teaching their partners	8
Rawat et al [15]	2022	Uganda	The study aimed to capture men’s knowledge, beliefs and perspectives about cervical cancer, community-based screening and health system barriers	23 men	Qualitative survey	Men’s knowledge Education of men and women Affordable transport Distance to facilities	7
Ngwenya and Huang [20]	2018	Eswatini	The study intended to explore the knowledge, attitudes and practice on cervical cancer and screening	202 men and 213 women	Quantitative Cross sectional	Knowledge and awareness Stigma beliefs Cultural beliefs	7
Williams & Amoateng [21]	2012	Ghana	The study aimed to assess Ghanaian men’s knowledge and beliefs about cervical cancer risk factors and cervical cancer screenings, and to identify cultural beliefs and practices that may lead to a lack of spousal support for cervical cancer screening; and to assess Ghanaian men’s willingness to encourage their spouses to get screened for cervical cancer	29 men	Qualitative Cross-sectional	Inaccurate knowledge Stigma beliefs Cultural taboos	6
Binka et al [22]	2019	Ghana	The study explored male knowledge and support during cervical cancer screening and treatment in a rural setting in Ghana	41 male partners and cervical cancer patients	Qualitative Cross-sectional	Lack of knowledge Support during screening and treatment	5
Rosser et al [23]	2014	Kenya	The study intended to understand the role of male partner support in women’s utilization of screening and treatment	110 men	Quantitative Cross-sectional	Awareness and Knowledge Stigma Perception of risk Intention to screen	7
Adewumi et al [25]	2019	Kenya	The study examined the role of male partners and community leaders in decision-making and accessing screening services	604 women	Qualitative Cross sectional	Decision making Screen and treatment Influence of male partners	6
Lewis et al [24]	2020	Malawi	The study’s aim was to evaluate married Malawian men on their knowledge and beliefs about cervical cancer	120 men	Qualitative Cross-sectional	Knowledge Female partner’s decision Screening process	5
Okedo-Alex et al [16]	2020	Nigeria	This study assessed married men´s knowledge and attitude towards male involvement in cervical cancer screening of their wives	245 men	Mix methods Cross-sectional	Inadequate knowledge Risk factors Willing to approve screening for partner Screening is important Willing to pay	7
Okafor et al [17]	2023	Nigeria	The study assessed male involvement in their female partners’ screening for breast and cervical cancers in Southwest	254 men	Cross-sectional	Knowledge Positive attitude Financial support Accompany partner	8
Ongtengco et al [18]	2020	Senegal	The study analysed gender differences on perceptions of gender roles, discrimination, cancer attitudes, cancer stigma, and influences in healthcare decision making	101 women and 57 men	Quantitative Cross-sectional	Education level Marital status Ever screened Community beliefs	5
Erin et al [19]	2018	Uganda	This preliminary study explores Ugandan men’s knowledge and attitudes about human papillomavirus (HPV), cervical cancer, and screening	62 men	Randomised control trails educational session	Knowledge about HPV virus Support partner to screen Vaccinate daughters Ongoing Education	4
De Fouw et al [26]	2023	Uganda	The study aimed to understand the perspectives of males on cervical cancer screening and HPV vaccination in Western-Uganda	67 men	Qualitative Cross-sectional	Stigmatization and family problems due to loss of fertility less marital sexual activity Support partners to screen Encourage daughters to get vaccinated	7
Nyambe [31]	2019	Zambia	The study investigated the social economic model and the theory of triadic influence in relation to cervical cancer prevention	300 women and 300 men	Cross-sectional \mixed methods	Causes and prevention of cervical cancer Knowledge and awareness Interpersonal interactions vaccination of daughters	4

### Study Findings Based on the Above Table

Several studies across various African countries have explored the knowledge, attitudes, and involvement of men regarding cervical cancer screening and prevention for their female partners. These studies employed quantitative, qualitative and mixed methods approaches to understand the role of men in influencing women’s utilization of cervical cancer screening services and HPV vaccination for daughters. The studies aimed to identify factors that encourage or hinder male involvement in cervical cancer prevention efforts.

In Nigeria, studies by Chigozie et al. (2021) and Okedo-Alex et al. (2020) investigated men’s willingness to encourage and financially support their family members for cervical cancer screening and HPV vaccination. These studies found that men’s perception of HPV risk, education level, income, and knowledge about cervical cancer were significant predictors of their willingness to be involved in these preventive measures. In South Africa, studies by Rwamugira et al. (2019) and Maree et al. (2011) focused on empowering men to educate their female partners about cervical cancer and motivate them to undergo screening. These studies revealed that men’s knowledge about HPV infection, cervical cancer, and screening programmes, as well as their cultural beliefs and education levels, played a crucial role in their involvement in cervical cancer prevention efforts.

Studies in Uganda by Rawat et al. (2022), Erin et al. (2018), and De Fouw et al. (2023) explored men’s perspectives on cervical cancer screening, HPV vaccination, and associated stigma. These studies found that men’s knowledge about HPV and cervical cancer, as well as their willingness to support partners in screening and vaccinate daughters, were influenced by factors such as education, affordability of transport, distance to facilities, and cultural beliefs about fertility and sexual activity. In Ghana, Williams and Amoateng (2012) and Binka et al. (2019) examined men’s knowledge, beliefs, and support for cervical cancer screening. These studies highlighted the prevalence of inaccurate knowledge, stigma beliefs, and cultural taboos as barriers to men’s involvement in supporting their partners’ screening efforts. However, men expressed a willingness to encourage their partners if provided with accurate information and education about cervical cancer and screening.

In summary, these studies across various African countries consistently identified men’s knowledge, education level, cultural beliefs and perception of risk as critical factors influencing their involvement in cervical cancer prevention efforts for their female partners and daughters. Addressing these factors through education, awareness campaigns and community engagement could potentially increase male involvement and support for cervical cancer screening and HPV vaccination programmes.

## Discussion

The objective of this research was to provide empirical data that may inform decision-making and policy interventions aimed at enhancing programmes and initiatives to facilitate the eradication of cervical cancer in Africa. The second point to consider is that the objective of this study is to assess the prevalence of cervical cancer screening uptake and evaluate the influence of male participation in the screening process among African women. African males exhibit the characteristics of a collective society, and the results of this study may provide researchers and practitioners with a valuable viewpoint to inform interventions and suggestions for African households in relation to cervical cancer screening. Understanding the factors that influence cervical cancer screening among African women is crucial for developing effective interventions. By evaluating the influence of male participation in the screening process, this study aimed to shed light on the role of African men in promoting women’s health. This research may provide valuable insights into the design of culturally sensitive interventions that could increase cervical cancer screening rates and ultimately reduce the burden of the disease in Africa.

This review yielded a number of significant results in relation to this matter. Although African males have little awareness of cervical cancer screening procedures, they are willing to assist their partners in completing the screening process. Additionally, they suggested several tactics that could be used to provide help in this regard. This finding is consistent with the results of a study conducted among men in Ghana. The men said they would help their partners get screened for cervical cancer if they knew a lot about the disease and the right way to perform the screening [21]. Research conducted among men in Uganda revealed that there is a significant desire among men to learn more about cervical cancer to facilitate their spouses’ engagement in health care-seeking behaviours [19]. These findings suggest that there is growing recognition among African men regarding the importance of cervical cancer screening and their role in supporting their partners’ health. However, it is crucial to ensure that these men have access to accurate and comprehensive information about cervical cancer and screening methods to effectively contribute to their partners’ healthcare decisions. Additionally, targeted educational programmes can be developed to address the specific knowledge gaps identified in these studies and empower men to actively participate in promoting cervical cancer prevention and early detection.

According to several philosophical perspectives, male partners assume the role of guardians in relation to the wellbeing of their spouses [32]. In such situations, the limited understanding of risk factors and preventive measures for cervical cancer might be a notable obstacle for men seeking to actively engage and provide support. According to research conducted on married men, there is a direct correlation between the adoption of behaviours that promote cervical cancer screening for female partners and the level of awareness about cervical cancer among their male counterparts [33]. This underscores the need to possess comprehensive and pertinent information and awareness of cervical cancer [33]. Without this knowledge, men may not fully understand the importance of cervical cancer screening, and may not actively support their female partners in seeking preventive measures. Furthermore, a lack of awareness of cervical cancer can lead to misconceptions and stigma, further hindering men’s involvement in promoting screening and support for their partners. Addressing this knowledge gap through educational initiatives tailored to men’s needs and cultural contexts could play a crucial role in empowering them to become advocates for cervical cancer prevention within their families and communities.

Most men expressed that their support is important for their partners to maintain good health as individuals and households. They believed that actively supporting their partners could help them make healthier choices and encourage positive habits. Additionally, these men understand that their support not only benefits their partners’ physical health, but also contributes to their overall wellbeing and happiness. African men are willing to provide emotional support to help their partners manage the potential uneasiness of cervical cancer screening and are willing to go along with their partners on cervical cancer screening schedules, attend appointments with them, and offer reassurance throughout the process. The extent of their engagement indicates a profound dedication to their partners’ wellbeing and exemplifies the significance they attribute to maintaining transparent communication and trust within their relationship. African men play a crucial role in mitigating the stigma associated with cervical cancer and advocating for preventive healthcare within their communities by actively participating in cervical cancer screening. By leveraging the willingness of African men to support their partners and their recognition of the importance of communication and trust, cervical cancer prevention programs could be designed to involve men as allies and advocates, promoting a more supportive and inclusive environment for women to seek screening services.

One of the most common obstacles to cervical cancer screening is financial constraints. Therefore, providing assistance to alleviate these barriers has the potential to enhance women’s participation in cervical cancer screening. Considering the evident inclination of African males to provide support for their spouses in accessing cervical cancer screening, it is imperative that forthcoming initiatives include the active involvement of male partners. To enhance the efficacy of health education and awareness initiatives pertaining to women’s health, it is essential to include and engage men, thus reinforcing male partners’ involvement in the uptake and prevention of cervical cancer screening. Research endeavours, including the community, should prioritise a family-oriented approach rather than an individual-centric approach, with the aim of shaping the collective culture of the African community to emphasise this significant attribute. Involving men in women’s health education can foster a supportive environment in which both partners understand the importance of cervical cancer screening. This family-oriented approach will not only benefit women, but also contribute to the overall wellbeing of the African community by promoting a culture of shared responsibility for healthcare.

There is a need for decentralization of services targeting male partners in conjunction with community outreach efforts for cervical cancer screening. The active involvement of men in the execution, dissemination, and strategic development of community-oriented cervical cancer screening initiatives is of the utmost importance. Failing to include males is a lost opportunity to provide them with access to various services and educational opportunities. In low-income settings, it is essential to implement coordinated initiatives aimed at educating males and addressing health system obstacles to facilitate cervical cancer screening and subsequent treatment of women. These initiatives should be tailored to the specific cultural, socioeconomic, and geographic contexts of the target populations, as barriers and facilitators may vary across different regions and communities. To enhance the adoption of cervical screening, it is advisable to customise the approach to align with the specific cultural context, literacy levels, and prevailing attitudes of the target population. By tailoring the approach to cultural context, literacy levels, and prevailing attitudes, it is more likely that the target population will be receptive to cervical screening. Conducting community outreach initiatives, collaborating with neighbourhood leaders and organizations, and distributing educational materials in a way that the public can understand could all be part of this. Additionally, addressing health system obstacles, such as limited resources and a lack of trained personnel, is crucial to ensuring the successful implementation of cervical cancer screening programmes in low-income settings.

### Implications for Practice

These findings have significant implications for the design and implementation of cervical cancer screening programs in African countries. Firstly, it is crucial to recognise the influential role that male partners can play in promoting women’s participation in screening services. Rather than treating cervical cancer screening as an individual responsibility, a family-oriented approach that actively involves and empowers men as allies and advocates could be more effective in addressing cultural and social barriers.

To achieve this, healthcare providers and community outreach initiatives should prioritise the inclusion of men in educational efforts related to cervical cancer prevention. By tailoring these educational programs to address the specific knowledge gaps and misconceptions prevalent among African men, they can be equipped with accurate information about the importance of screening, risk factors, and the screening process itself. This knowledge can empower men to support their partners’ decision to undergo screening and encourage open communication within the household.

Additionally, healthcare facilities and screening services should strive to create a welcoming and inclusive environment for male partners. Providing opportunities for men to accompany their partners during appointments, offering educational resources tailored to their needs, and training healthcare staff to engage with male partners respectfully can contribute to a more supportive atmosphere. By fostering a sense of involvement and shared responsibility, men may be more inclined to actively participate in their partners’ healthcare decisions and facilitate access to screening services.

### Implications for Future Research

While this review has shed light on the potential impact of male involvement in cervical cancer screening among African women, there is a need for further research to address remaining gaps and inform more effective interventions. Firstly, future studies should explore the specific cultural, social, and economic factors that influence male attitudes and involvement in cervical cancer screening across different regions and communities within Africa. As the findings of this review suggest, there may be variations in cultural beliefs, gender norms, and socioeconomic circumstances that shape men’s perspectives and ability to support their partners’ health.

By conducting in-depth qualitative and mixed-methods research within diverse African communities, researchers can gain a more nuanced understanding of the barriers and facilitators to male involvement. This knowledge can inform the development of tailored interventions that address the unique needs and challenges of each community, rather than adopting a one-size-fits-all approach.

Furthermore, longitudinal studies and intervention-based research are needed to evaluate the long-term impact of engaging male partners in cervical cancer screening initiatives. While the existing literature highlights the potential benefits of male involvement, there is a lack of evidence on the sustained effects of such interventions on screening uptake, adherence, and ultimately, cervical cancer outcomes. By conducting rigorous evaluations of pilot programs and community-based interventions that actively involve men, researchers can generate evidence to inform scalable and effective strategies for cervical cancer prevention in African contexts.

Additionally, future research should explore the potential of leveraging existing community structures and resources to facilitate male engagement in cervical cancer screening. This could include collaborating with religious institutions, traditional leaders, or community-based organizations that have established trust and influence within African communities. By integrating cervical cancer education and male involvement initiatives within these existing networks, researchers may be able to reach a broader audience and leverage the cultural and social capital of these institutions.

### Limitations

This review is limited to Africa. The studies were limited due to the exclusion criterion of French language articles. As countries covering the sub-Saharan Africa, part of them are French speaking countries like Burundi, D.R.Congo, Congo and others, many of their scientific articles are also in French.

Strengths: This systematic review could help African governments and NGOs to run an implementation research and health policies in decision-making to eradicate the cervical cancer in Africa as the vaccines exist but not affordable in terms of cost effectiveness and availability. The findings have positive global implications.

### Conclusion

One significant and conclusive conclusion derived from this research pertains to the endorsement of a joint decision-making paradigm by a majority of male participants. African men have varied perspectives on the need for their spouses to obtain consent prior to undergoing cervical cancer screening. Most male individuals expressed a preference for fostering collaborative or reciprocal decision-making processes for proactive healthcare services, specifically for cervical cancer screening. Conversely, a subset of individuals indicated a preference for their partners to independently make these choices while maintaining open lines of communication to remain informed. Prior research has shown that engaging in shared decision-making between couples may lead to more favourable health outcomes compared to situations in which males make choices in isolation or women make decisions without considering or obtaining consent from their partners [34]. This highlights the importance of involving both partners in the decision-making process for cervical cancer screening. By engaging in shared decision-making, couples can ensure that both perspectives are considered, leading to more informed choices and, ultimately, better health outcomes. This approach fosters a sense of mutual respect and equal involvement in healthcare decisions, thereby promoting a healthier and more equitable relationship. Additionally, maintaining open lines of communication allows for ongoing support and understanding, creating a solid foundation for addressing concerns or fears related to cervical cancer screening.

The findings of this research have significant implications for the development of culturally sensitive and gender-inclusive interventions aimed at increasing cervical cancer screening rates in African countries. By recognising the potential influence of male partners and involving them as active participants in the decision-making process and educational initiatives, these interventions can leverage the cultural values of collective responsibility and shared decision-making within African communities. Furthermore, the endorsement of shared decision-making by African men highlights the need for cervical cancer prevention programs to adopt a family-centred approach, where both partners are empowered with knowledge and engaged in the process. Ultimately, fostering an environment of mutual understanding, open communication, and shared responsibility between partners could lead to more informed decisions and higher uptake of cervical cancer screening services, contributing to the goal of reducing the burden of this disease in Africa.

## References

[1] IARC and WHO. GLOBOCAN 2018: Estimated Cancer Incidence, Mortality and Prevalence Worldwide in 2018. Cervical Cancer Fact Sheet (2018).

[2] World health Organization. Global Strategy to Accelerate the Elimination of Cervical Cancer as a Public Health Problem (2020).

[3] Arbyn M Weiderpass E Bruni L de Sanjosé S Saraiya M Ferlay J Estimates of Incidence and Mortality of Cervical Cancer in 2018: A Worldwide Analysis. Lancet Glob Health (2020) 8(2):e191–e203. 10.1016/S2214-109X(19)30482-6 31812369 PMC7025157

[4] World Health Organization. Comprehensive Cervical Cancer Control: A Guide to Essential Practice. 2nd ed. Geneva, Switzerland (2014). 25642554

[5] WHO. Comprehensive Cervical Cancer Prevention and Control E A Healthier Future For Girls And Women Geneva, Switzerland (2013). Available from: http://www.who.int/reproductivehealth/publications/cancers/9789241505147/en/(Accessed August 25, 2020).

[6] CDC. Gynecologic Cancer Awareness (2017). Available from: https://www.cdc.gov/cancer/dcpc/resources/features/gynecologiccancers/index.htm (Accessed November 23, 2017).

[7] Baskaran P Subramanian P Rahman RA Ping WL Taib NAM Rosli R. Perceived Susceptibility, and Cervical Cancer Screening Benefits and Barriers in Malaysian Women Visiting Outpatient Clinics. Asian Pac J Cancer Prev APJCP (2013) 14(12):7693–9. 10.7314/apjcp.2013.14.12.7693 24460355

[8] Leung SS Leung I. Cervical Cancer Screening: Knowledge, Health Perception and Attendance Rate Among Hong Kong Chinese Women. Int J Wom Health (2010) 2:221–8. 10.2147/ijwh.s10724 PMC2971734 21072314

[9] Busingye P Nakimuli A Nabunya E Mutyaba T. Acceptability of Cervical Cancer Screening via Visual Inspection With Acetic Acid or Lugol's Iodine at Mulago Hospital, Uganda. Int J Gynecol Obstet (2012) 119(3):262–5. 10.1016/j.ijgo.2012.06.015 22980432

[10] Birhanu Z Abdissa A Belachew T Deribew A Segni H Tsu V Health Seeking Behavior for Cervical Cancer in Ethiopia: A Qualitative Study. BMC Int J Equity Health (2012) 11(83):83. 10.1186/1475-9276-11-83 PMC3544623 23273140

[11] Wondimu YT. Cervical Cancer: Assessment of Diagnosis and Treatment Facilities IN Public Health Institutions IN Addis Ababa, Ethiopia. Ethiop Med J (2015) 13(2). 26591294

[12] Page MJ McKenzie JE Bossuyt PM Boutron I Hoffmann TC Mulrow CD The PRISMA 2020 Statement: An Updated Guideline for Reporting Systematic Reviews. bmj (2021) 372:n71. 10.1136/bmj.n71 33782057 PMC8005924

[13] Babineau J. Product Review: Covidence (Systematic Review Software). J Can Health Libraries Association/Journal de l'Association des bibliothèques de la santé du Can (2014) 35(2):68–71. 10.5596/c14-016

[14] Luchini C Stubbs B Solmi M Veronese N. Assessing the Quality of Studies in Meta-Analyses: Advantages and Limitations of the Newcastle Ottawa Scale. World J Meta-Analysis (2017) 5(4):80–4. 10.13105/wjma.v5.i4.80

[15] Rawat A Mithani N Sanders C Namugosa R Payne B Mitchell-Foster S We Shall Tell Them With Love, Inform Them What We Have Learnt and Then Allow Them to Go - Men's Perspectives of Self-Collected Cervical Cancer Screening in Rural Uganda: A Qualitative Inquiry. J Cancer Educ (2023) 38(2):618–24. 10.1007/s13187-022-02163-x 35384556

[16] Okedo-Alex IN Uneke CJ Uro-Chukwu HC Akamike IC Chukwu OE. It Is what I Tell Her that She Will Do: A Mixed Methods Study of Married Men's Knowledge and Attitude Towards Supporting Their Wives' Cervical Cancer Screening in Rural South-East Nigeria. Pan Afr Med J (2020) 36:156. 10.11604/pamj.2020.36.156.21157 32874420 PMC7436653

[17] Okafor IP Kukoyi FO Kanma-Okafor OJ Izuka MO. Male Involvement in Female Partners’ Screening for Breast and Cervical Cancers in Southwest Nigeria. PLoS One (2023) 18(5):e0284141. 10.1371/journal.pone.0284141 37163507 PMC10171596

[18] Ongtengco N Thiam H Collins Z De Jesus EL Peterson CE Wang T Role of Gender in Perspectives of Discrimination, Stigma, and Attitudes Relative to Cervical Cancer in Rural Sénégal. PLoS One (2020) 15(4):e0232291. 10.1371/journal.pone.0232291 32343755 PMC7188246

[19] Moses E Pedersen HN Wagner EC Sekikubo M Money DM Ogilvie GS Understanding Men's Perceptions of Human Papillomavirus and Cervical Cancer Screening in Kampala, Uganda. J Glob Oncol (2018) 4:1–9. 10.1200/JGO.17.00106 PMC6223419 30241236

[20] Ngwenya D Huang SL. Knowledge, Attitude and Practice on Cervical Cancer and Screening: A Survey of Men and Women in Swaziland. J Public Health (Oxf) (2018) 40(3):e343–50. 10.1093/pubmed/fdx174 29294055

[21] Williams MS Amoateng P. Knowledge and Beliefs About Cervical Cancer Screening Among Men in Kumasi, Ghana. Ghana Med J (2012) 46(3):147–51. 23661828 PMC3645156

[22] Binka C Doku DT Nyarko SH Awusabo-Asare K. Male Support for Cervical Cancer Screening and Treatment in Rural Ghana. PLoS One (2019) 14(11):e0224692. 10.1371/journal.pone.0224692 31738796 PMC6860429

[23] Rosser JI Zakaras JM Hamisi S Huchko MJ. Men's Knowledge and Attitudes About Cervical Cancer Screening in Kenya. BMC Womens Health (2014) 14:138. 10.1186/s12905-014-0138-1 25416335 PMC4254217

[24] Lewis S Moucheraud C Schechinger D Mphande M Banda BA Sigauke H A Loving Man Has a Very Huge Responsibility: A Mixed Methods Study of Malawian Men's Knowledge and Beliefs about Cervical Cancer. BMC Public Health (2020) 20(1):1494. 10.1186/s12889-020-09552-1 33008344 PMC7532091

[25] Adewumi K Oketch SY Choi Y Huchko MJ. Female Perspectives on Male Involvement in a Human-Papillomavirus-Based Cervical Cancer-Screening Program in Western Kenya. BMC Womens Health (2019) 19(1):107. 10.1186/s12905-019-0804-4 31395060 PMC6688365

[26] De Fouw M Stroeken Y Niwagaba B Musheshe M Tusiime J Sadayo I Involving Men in Cervical Cancer Prevention; A Qualitative Enquiry into Male Perspectives on Screening and HPV Vaccination in Mid-western Uganda. PLoS One (2023) 18(1):e0280052. 10.1371/journal.pone.0280052 36706114 PMC9882699

[27] Chigozie N Hilfinger Messiaa DK Adebola A Ojiegbe T. Men's Willingness to Support HPV Vaccination and Cervical Cancer Screening in Nigeria. Health Promot Int (2022) 37(1):daab056. 10.1093/heapro/daab056 33993249

[28] Rwamugira J Maree JE Mafutha N. The Knowledge of South African Men Relating to Cervical Cancer and Cervical Cancer Screening. J Cancer Educ (2019) 34(1):130–6. 10.1007/s13187-017-1278-4 28879562

[29] Maree JE Wright SC Makua TP. Men's Lack of Knowledge Adds to the Cervical Cancer Burden in South Africa. Eur J Cancer Care (Engl) (2011) 20(5):662–8. 10.1111/j.1365-2354.2011.01250.x 21501266

[30] Rwamugira J Maree JE Mafutha N. The Outcomes of an Educational Program Involving Men as Motivators to Encourage Women to Be Screened for Cervical Cancer. J Cancer Educ (2019) 34(2):269–76. 10.1007/s13187-017-1297-1 29139071

[31] Nyambe A Kampen JK Baboo SK Van Hal G. Knowledge, Attitudes and Practices of Cervical Cancer Prevention Among Zambian Women and Men. BMC Public Health (2019) 19(1):508. 10.1186/s12889-019-6874-2 31054569 PMC6500583

[32] World Health Organization. Comprehensive Cervical Cancer Control: A Guide to Essential Practice. Geneva: World Health Organization (2006). 25642554

[33] Suzu CA Elizabeth A-O Adejumo A. Husbands’knowledge, Attitude and Behavioural Disposition to Wives Screening for Cervical Cancer in Ibadan. Afr J The Psychol Stud Social Issues (2014) 17(2):167–76.

[34] Rao N Esber A Turner A Chilewani J Banda V Norris A. The Impact of Joint Partner Decision Making on Obstetric Choices and Outcomes Among Malawian Women. Int J Gynecol & Obstet (2016) 135(1):61–4. 10.1016/j.ijgo.2016.03.019 PMC5881379 27357611

