# Supplementary material for: “A Man With a Loving Heart”: A Systematic Review of Male Involvement in Cervical Cancer Screening in Africa
Source: Int J Public Health. 2024 Oct 10;69:1607447. doi: 10.3389/ijph.2024.1607447 (PMC11499092; doi:10.3389/ijph.2024.1607447)
Supplement: Supplementary file 1 [file Table1.docx]

**Appendix A. Supplementary data**

Quality and country of included studies supplementary materials (Table 2)

**Appendix A: Table 2. Supplementary materials quality and country of included studies. January 2010 to December, Africa 2023.**

| **Study** | **Country** | **Quality** | | | |
| --- | --- | --- | --- | --- | --- |
|  |  | Selection | Comparability | Outcome | Total score |
| Chigozie et al [14] | Nigeria | *** | ** | ** | 8 |
| Rwamugira et al [15] | South Africa | **** | ** | ** | 7 |
| Maree et al [16] | South Africa | *** | ** | ** | 8 |
| Rwamugira et al [17] | South Africa | **** |  | * | 8 |
| Rawat et al [18] | Uganda | **** | * | * | 7 |
| Ngwenya and Huang [19] | Eswatini | *** | ** | ** | 7 |
| Williams & Amoateng [20] | Ghana | **** | ** | ** | 6 |
| Binka et al [21] | Ghana | **** | ** | ** | 5 |
| Rosser et al [22] | Kenya | **** | ** | ** | 7 |
| Adewumi et al [23] | Kenya | **** | ** | ** | 6 |
| Lewis et al [24] | Malawi | **** | ** | ** | 5 |
| Okedo-Alex et al [25] | Nigeria | **** | ** | ** | 7 |
| Okafor et al [26] | Nigeria | **** | ** | ** | 8 |
| Ongtengco et al [27] | Senegal | **** | ** | ** | 5 |
| Erin et al [28] | Uganda | *** | ** | ** | 4 |
| De Fouw et al [29] | Uganda | *** | * | * | 7 |
| Nyambe [30] | Zambia | **** | ** | ** | 4 |
